# Comparative meta-analysis of vertebral body tethering and posterior spinal fusion in patients with idiopathic scoliosis. Evaluation of radiographic, perioperative, clinical, patient-reported outcomes, and complication rates

**DOI:** 10.1007/s43390-025-01113-z

**Published:** 2025-06-08

**Authors:** Stavros Stamiris, Cornelius Sofos, Athanasios Sarridimitriou, Panagiotis Kakoulidis, Panagiotis Christidis, Dimitrios Stamiris, Elissavet Anestiadou, Angeliki Cheva, Christiana Chatzianestiadou, Pavlos Christodoulou, Christos Karampalis

**Affiliations:** 1 Department of Orthopaedics, 424 General Military Hospital, West Ring Road, Nea Efkarpia, 56429 Thessaloniki, Greece; 2 3rd Academic Department of Orthopaedics, Faculty of Medicine, Aristotle University of Thessaloniki, West Ring Road, Nea Efkarpia, 56429 Thessaloniki, Greece; 3 Department of Trauma and Orthopaedics, Frimley Health NHS Foundation Trust, Camberley, UK; 4 4th Academic Department of Surgery, Faculty of Medicine, Aristotle University of Thessaloniki, Thessaloniki, Greece 54124; 5 Department of Pathology (Laboratory of General Pathology and Pathological Anatomy), Faculty of Medicine, Aristotle University of Thessaloniki, Thessaloniki, Greece; 6 Faculty of Medicine, Aristotle University of Thessaloniki, Thessaloniki, Greece

**Keywords:** Spinal fusion, Vertebral body tethering, Idiopathic scoliosis, Meta-analysis

## Abstract

**Background:**

Although posterior spinal fusion (PSF) is considered the gold standard for the treatment of idiopathic scoliosis, it has been associated with several limitations. Vertebral body tethering (VBT) offers a motion-preserving alternative, with growing evidence supporting its clinical efficacy.

**Methods:**

A comprehensive search of PubMed, Cochrane, Web of Science and Scopus databases was performed to identify comparative studies between VBT and PSF in patients with idiopathic scoliosis. Primary outcomes included major curve correction and postoperative major and minor curve angles. Secondary outcomes included radiographic parameters (shoulder height difference, spinal height gain, coronal balance, thoracic kyphosis, lumbar lordosis), perioperative metrics [length of stay (LOS), estimated blood loss (EBL), operative time, instrumented levels], patient-reported outcomes [Scoliosis Research Society-22 Questionnaire (SRS-22)], complication and revision rates.

**Results:**

Seventeen studies met the inclusion criteria. VBT patients required shorter instrumentations (*p* < 0.00001). PSF achieved lower postoperative major (*p* < 0.00001) and minor curve angles (*p* = 0.00001), better coronal balance (*p* = 0.005) and superior major curve correction from baseline (*p* < 0.00001), but with questionable clinical significance. VBT demonstrated greater lumbar flexion capacity (*p* < 0.00001), superior shoulder balance (*p* < 0.00001) and better outcomes in SRS-22 pain (*p* = 0.02), satisfaction (*p* = 0.03) and function (*p* = 0.02) at two-year follow-up. VBT also had shorter operation times (*p* = 0.0007), less blood loss (*p* < 0.00001), but higher complication (*p* = 0.0002) and revision rates (*p* < 0.00001). No difference detected in lumbar lordosis (*p* = 0.08), thoracic kyphosis (*p* = 0.15), SRS-22 self-image (p = 0.20) and total (*p* = 0.12), lumbar side bending (*p* = 0.81), axial rotation (*p* = 0.43) and hospital stay (*p* = 0.7).

**Conclusions:**

PSF demonstrates superior coronal spinal alignment, along with lower complication and revision rates. In contrast, VBT offers better preservation of spinal motion, improved shoulder balance, enhanced early quality of life, and reduced blood loss and operative time, while requiring shorter instrumentations. Treatment decisions should be individualized, taking into account patient-specific factors. Long-term outcome data are needed to guide clinical practice.

**Supplementary Information:**

The online version contains supplementary material available at 10.1007/s43390-025-01113-z.

## Introduction

Spinal fusion is considered the gold standard for the surgical treatment of idiopathic scoliosis, providing reliable curve correction and trunk balance while preventing further progression. However, this technique also comes with notable drawbacks. The immobilization of instrumented vertebrae results in loss of spinal mobility [1] and increased stress on adjacent levels, which can lead to premature degenerative changes and long-term pain due to adjacent segment disease [2, 3]. Furthermore, fusion restricts spinal growth, making it less ideal for those with significant growth potential remaining [4]. To address these limitations, vertebral body tethering (VBT) has emerged as a motion-preserving, growth-modulating alternative. This technique utilizes a flexible tether on the convex side of the curve to correct spinal curvature while allowing further growth and preserving spinal mobility [5–7]. However, VBT also presents unique challenges, including the risk of tether breakage, overcorrection, and the potential need for revision surgery [8, 9]. The outcomes of VBT compared to posterior spinal fusion (PSF) remain under investigation, with emerging evidence suggesting differences in deformity correction, functional outcomes, patient satisfaction and complication rates.

This meta-analysis aims to systematically compare VBT and PSF in terms of curve correction, spinal alignment, trunk mobility, patient-reported outcomes, perioperative parameters, and complication rates. By synthesizing current evidence, we aim to provide a comprehensive assessment of the efficacy and safety of these two  techniques, guiding clinicians in selecting the optimal treatment for patients with idiopathic scoliosis.

## Materials and methods

### Guidelines followed

This meta-analysis followed the Preferred Reporting Items for a Systematic Reviews and Meta-analyses (PRISMA) guidelines [10] (PRISMA checklist is available in Supplementary Material 1) and was registered in the Prospective Register of Systematic Reviews (PROSPERO) (ID: CRD420250640371).

### Search strategy

In this meta-analysis, the Population, Intervention, Comparison, and Outcomes (PICO) model was implemented as follows: (i) Population: Patients with idiopathic scoliosis; (ii) Intervention: Patients treated with VBT; (iii) Comparison group: Patients treated with PSF; (iv) Outcomes: The primary outcomes were major and minor curve angle at final follow-up and major curve correction from baseline. Secondary outcomes included radiographic outcomes (coronal balance, thoracic kyphosis, lumbar lordosis, shoulder height difference, spinal height gain from baseline), postoperative trunk motion (flexion, extension, side bending and axial rotation), postoperative scores on the Scoliosis Research Society-22 Questionnaire (SRS-22), instrumented levels, operative time, length of stay (LOS), ​estimated blood​ ​loss​ (EBL), postoperative complications and revision rates.

The electronic databases of PubMed (MEDLINE), Cochrane (CENTRAL), Scopus and Web of Science were systematically searched from conception to February 5, 2025, to identify eligible studies. (Supplementary Material 2). The initial screening of studies based on title and abstract was completed independently by two investigators (SS and CS). The same reviewers assessed the full-text articles of eligible studies to make final inclusion decisions. A third investigator (DS) was consulted to resolve any discrepancies.

### Trial selection

Prior to conducting the literature search, specific inclusion and exclusion criteria were established. We included studies satisfying the following criteria: (i) comparative studies evaluating human patients with idiopathic scoliosis, treated surgically with VBT and PSF; (ii) studies reporting radiographic, patient-reported outcomes and complication rates with a minimum two-years follow-up period; (iii) studies reporting outcomes on trunk range of motion (ROM) and perioperative outcomes; and (iv) prospective or retrospective studies and randomized control trials.

Exclusion criteria were as follows: (i) studies investigating other surgical techniques or hybrid constructs; (ii) studies involving patients with previous spinal fracture, infection, malignancy or history of prior spine surgery; (iii) studies evaluating patients with scoliosis of non-idiopathic origin, including congenital, syndromic, or neuromuscular aetiology; (iv) studies reporting radiographic, patient reported outcomes and complications with a follow-up of less than two years.; (iv) non-human, cadaveric, animal or finite element analysis studies; (v) case series and case reports.

### Data extraction

All included studies were reviewed by two researchers (SS and CS). The following ​data were extracted and recorded: (i​) Research information: ​first author​, publication ​year, study’s country of origin and study design; (ii) Baseline characteristics: Lenke classification, study period, surgical technique used (e.g. open or arthroscopic), implant system and manufacturer, number of participants, age and follow-up duration; (iii) Radiographic outcomes: major and minor curve angles, major curve angle change from baseline (absolute or percentage), coronal balance, thoracic kyphosis, lumbar lordosis, shoulder height difference and spinal height gain from baseline; (iv) Functional outcomes: Trunk ROM (flexion, extension​, side bending, axial rotation); (v) Patient-reported outcomes: SRS-22 scores; (vi) Perioperative outcomes: Instrumented levels, LOS, EBL and operation time; and (vii) Adverse events: overall complication rates and revision rates. The patients in the included studies were divided into two groups based on the type of intervention they received.

### Imputation of data

In cases of missing data, a stepwise approach was adopted. Initially, we attempted to contact the corresponding authors to acquire the necessary information. If this was unsuccessful, appropriate statistical methods were employed to estimate the missing values and ensure a comprehensive analysis [11–13]. Finally, if missing data could not be addressed through these methods, the studies were excluded from the analysis of the outcome.

Specifically, standard deviations (SDs) were derived from ranges or p-value between groups, when available, using the methods proposed by Walter and Yao [11] and the Cochrane Handbook (Sect. 6.5.2.3) [13], respectively. When studies provided medians and interquartile ranges (IQRs), mean and SD values were estimated using Wan et al.'s approach [12]. For studies reporting means and SDs for subgroups, pooled values were calculated following Cochrane Handbook guidelines (Sect. 6.5.2.10) [13]. Finally, to address the variability in the reporting of ROM outcomes among the included studies, standardization was applied. Specifically, one study [14] reported side bending and axial rotation separately for left and right motion, whereas the other two [15, 16] presented total ROM for these movements. To ensure consistency, aggregate side bending and axial rotation in the first study were calculated by summing the mean values for left and right  motions, while the standard deviation (SD) was estimated using the variance sum law for correlated variables. Given that left and right side bending and axial rotation were reported as statistically symmetrical (*p* > 0.05), a correlation coefficient (*r* = 0.8) was assumed.

### Risk of bias and study quality assessment

**Fig. 1:**
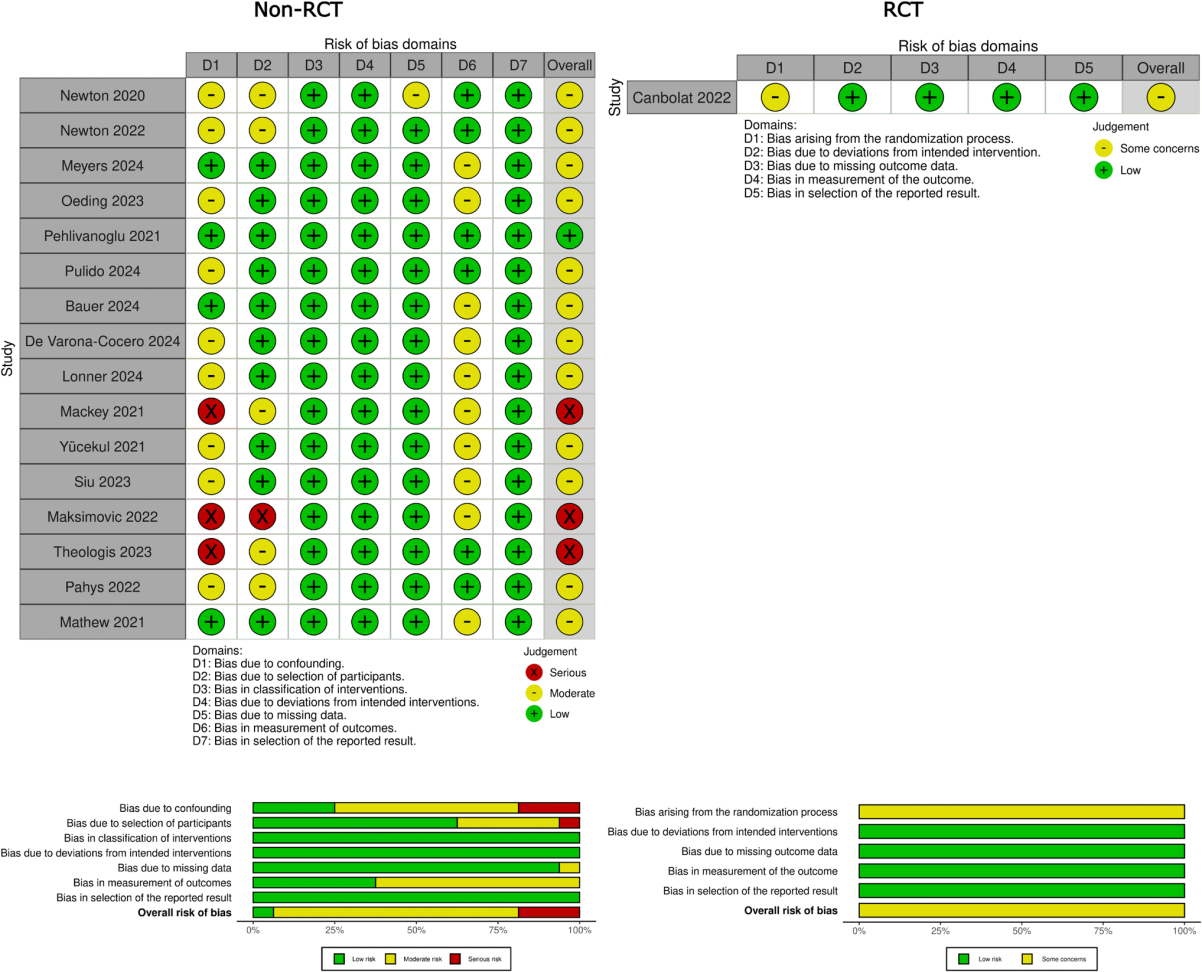
Risk of bias assessment for included studies using ROBINS-I for non-randomized studies and RoB 2.0 for the randomized controlled trial (RCT). Each domain is evaluated as low risk (green), some concerns (yellow), or serious risk (red). The summary graph at the bottom displays the overall risk distribution across domains

An independent quality assessment of the included studies was performed by the same two authors. For non-randomized trials, the risk of bias (RoB) was assessed using the revised Cochrane risk of bias in non-randomized studies of Interventions (ROBINS-I) tool [17], while for randomized controlled trials (RCTs), it was assessed using the revised Cochrane risk-of-bias tool for RCTs (RoB 2.0) [18]. The results of the RoB assessment for all included studies are summarised and presented in Fig. 1.

### Statistical analysis

All analyses were conducted using *Review Manager 5.4 software (Cochrane Library)*. For postoperative outcomes, if a significant preoperative difference existed between groups, propensity score-matched data were used when available; otherwise, the study was excluded to minimize bias. When multiple outcome measures were reported for the same parameter, those not requiring data imputation were preferred. Finally, thoracic kyphosis and lumbar lordosis were conducted separately for primary thoracic (Lenke 1–4) and lumbar curves (Lenke 5–6) to account for fundamental biomechanical differences. However, only studies focusing on Lenke 1–4 curves provided sufficient data for meta-analysis, while pooled analysis for Lenke 5–6 curves was not feasible due to limited studies.

Heterogeneity was assessed using Cochran’s Q test and the I^2^ statistic. I^2^ values were classified as low (< 25%), low to moderate (25–50%), substantial (50–75%) or considerable (> 75%). When I^2^ was between 25–50%, Cochran’s Q test p-value was examined; For *p* > 0.10, heterogeneity was considered likely due to random variation and classified as low, whereas *p* ≤ 0.10 suggested moderate heterogeneity. If heterogeneity exceeded low levels, the source was investigated, when possible, by conducting a sensitivity analysis, with rejection of the most influential study based on sample size and effect magnitude, or by performing subgroup analysis.

In cases with low heterogeneity, a fixed effects model was used, otherwise a random effects model was employed. Associations were reported as odds ratios (OR) and risk ratios (RR) with 95% confidence intervals (CI) for qualitative measurements and mean differences (MD ± standard errors) with 95% CI for quantitative data. For data reported in different scales across studies, the standardized mean difference (SMD) was employed to adjust for differences in measurement scales. Statistical significance was set at *p* < 0.05. Additional sensitivity analyses were performed to test the strength and validity of pooled results by the exclusion of studies with high risk of bias, to assess whether their occlusion had a disproportionate impact on the overall results. Publication bias was assessed by visual inspection of symmetry in funnel plots for outcomes reported in at least 10 studies [19].

## Results

### Literature search and selection process

**Fig. 2:**
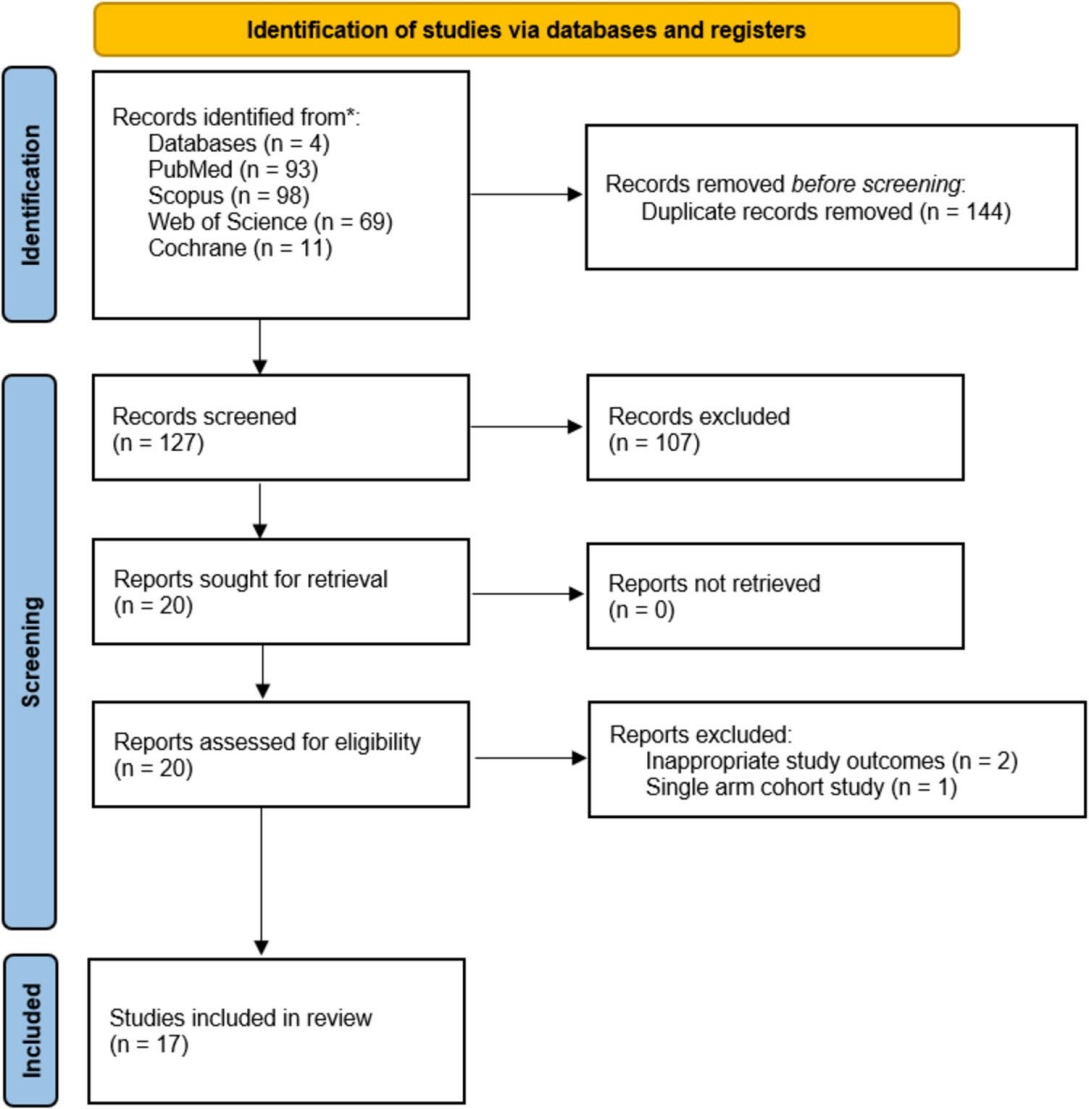
Preferred Reporting Items for Systematic Reviews and Meta-Analyses (PRISMA) flow diagram illustrating the study selection process. The figure outlines the number of records identified, screened, assessed for eligibility, and included in the final analysis

Our search strategy identified 271 potentially relevant studies. After the removal of duplicates (*n* = 144), 127 records were screened based on title and abstract. Full-text assessment was conducted for 20 studies, 3 of which were excluded for the following reasons: (i) one study reported outcomes only in the VBT group [20] (ii) two studies reported inappropriate outcomes (one study reported cost related outcomes [21] and one study reported pain and recovery speed related outcomes [22]). A flowchart diagram of the search strategy is shown in Fig. 2.

### Study characteristics

Seventeen studies published between 2020 and 2024 were finally included for qualitative and quantitative analysis, based on our pre-established criteria [14–16, 23–36]. The studies were conducted in the USA (*n* = 13), Turkey (*n* = 3), and one was a multicenter study (*n* = 1). Regarding study design, 16 were retrospective studies, one was a prospective study and one was a randomized control trial. All studies reported final outcomes with a minimum two-year follow-up, except for two [23, 25] which reported only perioperative outcomes. The total number of patients was 1549, with 761 in the VBT group and 788 in the PSF group. A summary of the descriptive characteristics of the included studies is presented in Table 1.

**Table 1:** Descriptive characteristics of the included studies

First Author, year	Country	Type of study	Lenke type curves	Study period (VBT/PSF)	Technique		Implant [Name (Manufacturer)]		Mean age		N (m/f)		Mean follow up time (years)
					VBT group	PSF group	VBT group	PSF group	VBT group	PSF group	VBT group	PSF group	
Newton et al. 2020	USA	Retrospective study	Lenke 1–4	2011–2016	AVBT	PSF	NR	NR	12 ± 2	13 ± 1	23 (7/16)	26 (3/23)	VBT 3.4 ± 1.1 PSF 3.6 ± 1.6
Newton et al. 2022	MC	Retrospective study	Lenke 1–4	2011–2018	AVBT	PSF	The Tether (Zimmer biomet) Reflect (Globus medical)	NR	12.1 ± 1.6	13.4 ± 1.4	237 (38/199)	237 (39/198)	VBT 2.2 ± 0.5 PSF 2.3 ± 0.5
Propensity score matching for age, preoperative thoracic curve magnitude, sex, and Risser sign									12.7 ± 1.6	12.9 ± 1.4	108 (13/95)	108 (14/94)	NR
Meyers et al. 2024	MC	Retrospective study	Lenke 1–2	NR	AVBT	PSF	NR	NR	12.9 ± 1.4	13.5 ± 2.2	46 (5/41)	45 (4/41)	NR
Lenke 2 subgroup											10	15	
Oeding et al. 2023	USA	Retrospective study	Lenke 1,3,6	2015–2019	ATVBT/PLST	PSF	The Tether (Zimmer biomet)	Stryker Xia (Stryker)	12.7 ± 1.4	12.7 ± 1.4	10 (0/10)	12 (0/12)	VBT 2.9 ± 0.9 PSF 2.5 ± 1.2
Pehlivanoglou et al. 2021	Turkey	Retrospective study	Lenke 1–4	2016–2019	Thoracoscopic VBT	PSF	NR	NR	11.1 ± 1.6	10.9 ± 1.6	21(15/6)	22 (6/16)	VBT 3.3 ± 0.3 PSF 3.3 ± 0.3
Pulido et al. 2024	USA	Retrospective study	NR	2018–2023/ 2016–2022	ATVBT/PLST	PSF	NR	NR	14.8 ± 2.1	13.3 ± 1.8	109 (19/90)	89 (13/76)	NR
Bauer et al. 2024	USA	Prospective study	Lenke 5,6	NR	Lumbar VBT	PSF	NR	NR	13.5 ± 1.5	14 ± 1.5	24	24	NR
De Varona-Cocero et al. 2024	MC—USA	Retrospective study	Lenke 1–3,5,6	NR	Mini-open thoracoscopic assisted, 2RVBT	PSF	NR	NR	13.6 ± 1.4	13.2 ± 1.9	49 (1/48)	50 (8/42)	NR
Propensity score matching for Lenke classification									12.9 ± 1.8	3.5 ± 1.5	27 (1/26)	27 (4/23)	
Lonner et al. 2024	USA	Retrospective study	Lenke 1,3,4–6	NR	VBT	PSF	NR	NR	13.1 ± 1.7	13.4 ± 1.6	29	29	VBT: 2.0 ± 0.2 PSF: 2.1 ± 0.4
Lenke 1–4 subgroup											17	17	NR
Lenke 5–6 subgroup											12	12	NR
Mackey et al. 2021	MC	Prospective study	NR	2013–2017/ 2007–2017	VBT	PSF	NR	NR	11.3 ± 0.7	10.9 ± 0.9	37 (1/36)	42 (8/34)	VBT 2.9 ± 1.2 PSF 3.1 ± 1.2
Yücekul et al. 2021	Turkey	Retrospective study	Lenke 1,2	2014–2019	Mini-open thoracoscopic assisted VBT	PSF	NR	NR	12.6 ± 1.4	14.4 ± 1.4	18 (1/17)	16 (3/13)	All 2.1 ± 0.2
Siu et al. 2023	USA	Retrospective study	Lenke 1	2014–2019	Open ATVBT	PSF	Dynesys system components, The Tether (Zimmer biomet)	NR	12 ± 1	13 ± 1	23 (3/20)	24 (2/22)	VBT 3.8 ± 1.6 PSF 3.3 ± 1.4
Canbolat et al. 2022	Turkey	RCT	NR	June–Nov 2021	Mini open VBT	PSF	NR	NR	13.5 ± 1.5	14.4 ± 1.8	14 (0/14)	17 (3/14)	NR
Maksimovic et al. 2022	USA	Retrospective study	Lenke 1–4	NR	AVBT	PSF	NR	NR	14 ± 1.7	16 ± 1.5	5	4	VBT 1.5 ± 0.6 PSF 2.7 ± 0.6
Theologis et al. 2023	USA	Retrospective study	Lenke 1–4	2013–2019	AVBT	PSF	The Tether (Zimmer biomet)	NR	14.2 ± 2.2	14.2 ± 2.2	25	78	NR
Pahys et al. 2022	USA	Retrospective study	Lenke 1–6	2016–2021	AVBT	PSF	NR	NR	13.2 ± 1.1	14 ± 1.8	65 (10/55)	47 (8/39)	NR
Mathew et al. 2021	USA	Prospective study with matched retrospective comparison group	Lenke 1–3,5	2016–2021	ATVBT ATVBT/PLST	PSF	Dynesys system components, The Tether (Zimmer biomet)	NR	13.2 ± 0.23	13.4 ± 0.2	26 (3/23)	26 (3/23)	VBT 2 ± 0.1 PSF 2.4 ± 0.1

Continuous data are displayed in Mean ± SD; *NR* not reported, *MC* multicenter, *PSF* posterior spinal fusion, *VBT* vertebral body tethering, *ATVBT* anterior thoracic vertebral body tethering, *PLST* posterior lumbar spinal tethering

### Major curve angle

**Fig. 3:**
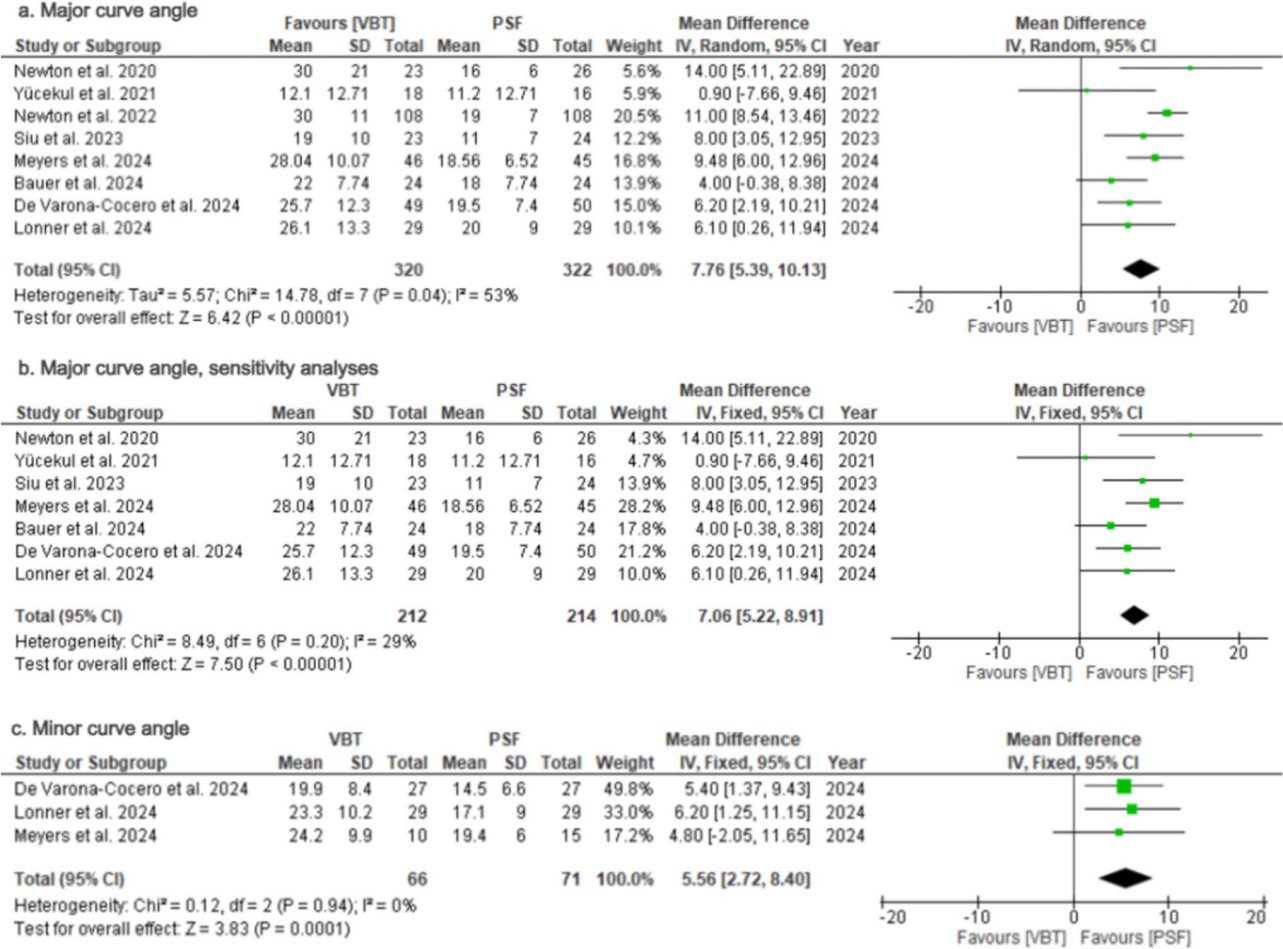
Forest Plots for major and minor curves outcomes. **a** Pooled analysis comparing major curve angle at the final follow-up between VBT and PSF. **b** Sensitivity analysis after exclusion of the study with the highest sample size. (c) Pooled analysis comparing minor curve angle at final follow-up between VBT and PSF

The major curve angle at the final follow-up was reported in eight studies [24, 26–28, 31, 35–37] (Table 2). Significant lower postoperative major curves were noted in the PSF group (MD = 7.76; 95%CI = 5.39–10.13; *p* < 0.00001; Fig. 3a), with substantial heterogeneity (*I*^2^ = 53%).

**Table 2:** Radiological outcomes

First Author, year	Major Cobb (^o^)		Major cobb correction (%)		Major cobb correction (^ο^)		Minor cobb (^o^)		Coronal balance (C7-CSVL) (cm)		Shoulder height difference (cm)		Spinal height gain (T1-T12) (mm)		Thoracic Kyphosis (T2-T12, T5-T12) (^o^)		Lumbar lordosis (^ο^)	
	VBT	PSF	VBT	PSF	VBT	PSF	VBT	PSF	VBT	PSF	VBT	PSF	VBT	PSF	VBT	PSF	VBT	PSF
Newton et al. 2020	30 ± 21	16 ± 6	43 ± 38	69 ± 13	NR	NR	NR	NR	1.3 ± 1	0.7 ± 0.5	0.7 ± 0.7	1.2 ± 1	NR	NR	19 ± 13^‡^	29 ± 8^‡^	NR	NR
Newton et al. 2022	**27 ± 12**	**20 ± 7**	43 ± 22	62 ± 13	−21 ± 39.5^a^	−33 ± 39.5^a^	NR	NR	NR	NR	0.7 ± 0.6	1 ± 1	NR	NR	19 ± 13^†^	21 ± 9*	57 ± 13	59 ± 13
PSM subgroup	30 ± 11	19 ± 7	41 ± 20	63 ± 13	−22 ± 24.2^a^	−33 ± 24.2^a^	NR	NR	NR	NR	NR	NR	NR	NR	NR	NR	NR	NR
Meyers et al. 2024	28 ± 10.1	18.6 ± 6.5	NR	NR	−19.4 ± 10.5	−31.6 ± 8.2	NR	NR	NR	NR	0.6 ± 0.5	0.9 ± 0.6	NR	NR	NR	NR	NR	NR
Lenke 2 subgroup	31.5 ± 15.3	19.1 ± 6.4	NR	NR	NR	NR	24.2 ± 9.9	19.4 ± 6.0	NR	NR	NR	NR	NR	NR	NR	NR	NR	NR
Pehlivanoglou et al. 2021	NR	NR	NR	NR	NR	NR	NR	NR	0.8 ± 0.5^d^	0.3 ± 0.2^d^	NR	NR	NR	NR	NR	NR	NR	NR
Bauer et al. 2024	22 ± 7.7^a^	18 ± 7.7^a^	NR	NR	NR	NR	NR	NR	2.8 ± 1.4^a^	1.1 ± 1.4^a^	NR	NR	NR	NR	NR	NR	55 ± 10.5^d^	68 ± 12.3^d^
De Varona et al. 2024	25.7 ± 12.3	19.5 ± 7.4	NR	NR	− 32.0 ± 11.3	− 37.2 ± 13.3	**18 ± 8.4**	**13.7 ± 6.3**	NR	NR	NR	NR	NR	NR	NR	NR	NR	NR
PSM subgroup	27.6 ± 13.2	19.9 ± 8.1	NR	NR	− 30.8 ± 11.8	− 38.9 ± 11.9	19.9 ± 8.4	14.5 ± 6.6	NR	NR	NR	NR	NR	NR	NR	NR	NR	NR
Lonner et al. 2024	26.1 ± 13.3	20 ± 9	50.4 ± 21.8	63.3 ± 14.8	−25.7 ± 12.9^c^	−33.7 ± 11.5^c^	23.3 ± 10.2	17.1 ± 9.0	NR	NR	NR	NR	NR	NR	23.8 ± 12.4^†^^,^^c^	24.78 ± 9.1^†^^,^^c^	59.2 ± 13^c^	64.1 ± 13^c^
Lenke 1–4 subgoup	31.6 ± 12.0	17.4 ± 6.5	39.9 ± 16.8	68.3 ± 10.6	−19.9 ± 11.8^a^	−36.9 ± 11.8^a^	25.3 ± 10.0	15.8 ± 7.0	NR	NR	NR	NR	NR	NR	24.4 ± 12.5^†^	27.1 ± 7.4^†^	59.9 ± 13.2	61.9 ± 11.6
Lenke 5–6 subgroup	18.3 ± 11.4	23.8 ± 10.9	65.4 ± 19.5	56.2 ± 17.4	−34 ± 9.7^a^	−29.2 ± 9.7^a^	20.6 ± 10.3	18.9 ± 11.2	NR	NR	NR	NR	NR	NR	23 ± 12.8^†^	21.5 ± 10.5^†^	58.1 ± 13.1	67.2 ± 14.6
Mackey et al. 2021	**28 ± 10.8**^**b**^	**29 ± 10.8**^**b**^	41.1 ± 22.4	52.2 ± 19.9	NR	NR	NR	NR	NR	NR	NR	NR	43 ± 66.2^b^	50 ± 116^b^	**25.0 ± 13.0**^**‡**^	**25.8 ± 11.5**^**‡**^	NR	NR
Yücekul et al. 2021	12.1 ± 12.7^a^	11.2 ± 12.7^a^	76 ± 23^a^	80 ± 23^a^	NR	NR	NR	NR	NR	NR	NR	NR	NR	NR	28,1 ± 7^‡^	31,4 ± 10,8^‡^	56.1 ± 7.3	57.3 ± 9.5
Siu et al. 2023	19 ± 10	11 ± 7	63 ± 17	79 ± 15	NR	NR	NR	NR	0.3 ± 1.5	0.3 ± 1.7	0.7 ± 0.7	1 ± 0.9	NR	NR	22 ± 12^†^	18 ± 10^†^	NR	NR
Mathew et al. 2021	NR	NR	46 ± 23	66 ± 15	−23 ± 12	−34 ± 9	NR	NR	NR	NR	NR	NR	42 ± 20	26 ± 25	NR	NR	NR	NR

Continuous data are displayed in Mean ± SD; *NR* Not reported, Values in bold indicate a statistically significant difference in preoperative scores between groups

^†^T5-T12 measurement; ^‡^T2-T12 measurement

^a^SD was calculated form the mean and p between groups using the formula from the Cochrane handbook, Sect. 7.7.3.3

^b^Mean and SD were calculated from Median and IQR using the formula from the study by Wan, X. et al

^c^Combined mean and SD were calculated using the formula from the Cochrane handbook, Sect. 6.5.2.10

^d^SD was calculated from range and N using the formula from the study by Walter SD, Yao X

Sensitivity analysis with exclusion of one study [37] which had significantly larger sample size reduced heterogeneity to low (*I*^2^ = 29%; *p* = 0.2), while maintaining a similar effect size (MD = 7.06; 95%CI = 5.22–8.91; *p* < 0.00001; Fig. 3b), suggesting that the observed heterogeneity was primarily driven by the large sample size of this study rather than true clinical differences among studies.

### Minor curve angle

Three studies [24, 27, 28] reported outcomes on minor curve at the last follow-up (Table 2). Patients in the PSF group had a lower postoperative minor curve in comparison to the VBT group (MD = 5.56; 95%CI = 2.72–8.40; *p* = 0.0001; Fig. 3c) with no heterogeneity (*I*^2^ = 0%).

### Major curve correction from baseline

**Fig. 4:**
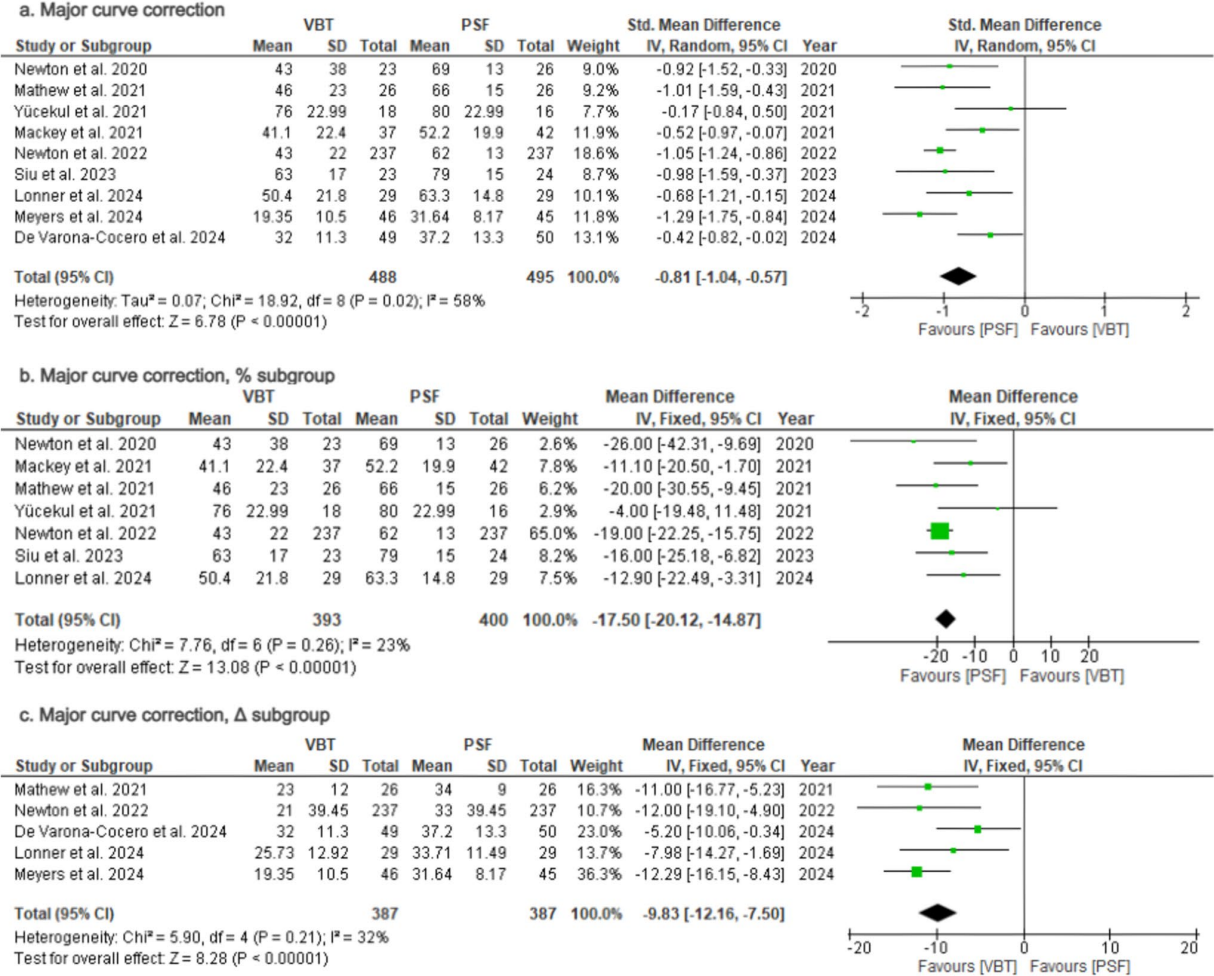
Forest Plots for Major Curve Correction. **a** Pooled analysis comparing major curve correction from baseline between VBT and PSF. **b** Subgroup analysis for major curve correction as a percentage change from baseline. **c** Subgroup analysis for major curve correction as absolute change (Δ) from baseline

Nine studies [24, 27, 28, 30, 31, 33–36] reported major Cobb correction from baseline at last follow-up (Table 2). Patients in the PSF group showed greater improvement (SMD = −0.81; 95%CI = −1.04–−0.57; *p* < 0.00001; Fig. 4a), with substantial heterogeneity (*I*^2^ = 58%),

Subgroup analysis of seven studies [28, 30, 31, 33–36] reporting percentage change from baseline (%) and five studies [24, 27, 28, 30, 33] reporting absolute change from baseline (Δ) was performed to address heterogeneity. Both outcomes remained significant in favor of PSF group [(MD = −17.50; 95%CI = −20.12–−14.87; *p* < 0.00001; Fig. 4b) and (MD = −9.83; 95%CI = −12.16–−7.50; *p* < 0.00001; Fig. 4c) respectively], while heterogeneity was reduced to low [(*I*^2^ = 23%), (*I*^2^ = 32%; p = 0.21) respectively]. This suggests that variability in the original analysis was driven by differences in reporting methods, as absolute change (Δ) is influenced by baseline curve severity, while percentage change (%) normalizes for initial curve magnitude, reducing variability.

### Secondary outcomes

#### Radiological parameters

**Fig. 5:**
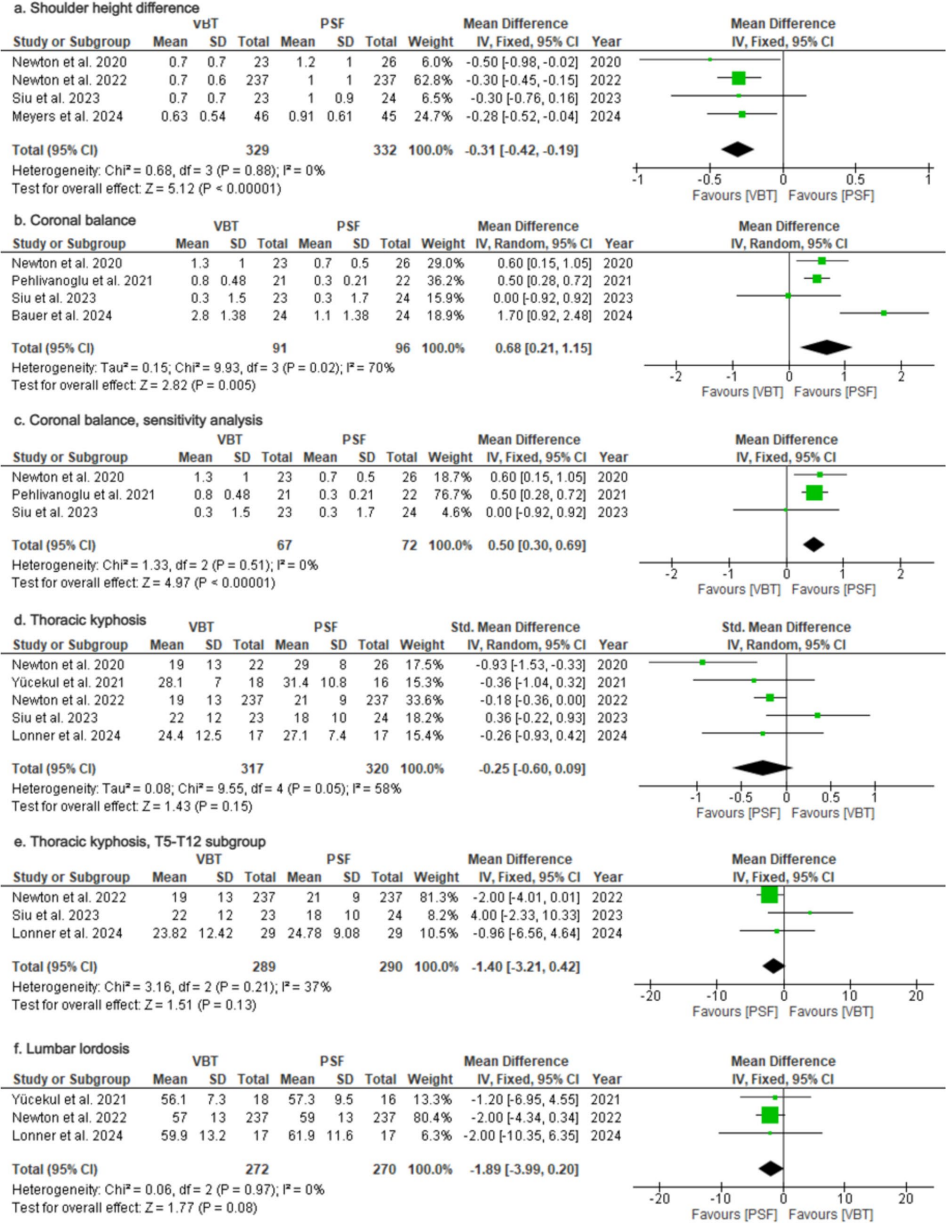
Forest Plots for Radiographic Outcomes **a** Pooled analysis comparing shoulder height difference between VBT and PSF. **b** Pooled analysis comparing coronal balance at final follow-up. **c** Sensitivity analysis for coronal balance. **d** Pooled analysis comparing thoracic kyphosis between VBT and PSF. **e** Subgroup analysis for thoracic kyphosis measured from T5-T12. **f** Pooled analysis comparing lumbar lordosis between VBT and PSF

*Shoulder height difference*: Four studies [27, 30, 31, 35] reported outcomes on shoulder height difference at final follow-up (Table 2). The VBT group had lower postoperative shoulder asymmetry compared to PSF group (MD = −0.31; 95%CI = −042–−0.19; *p* < 0.00001; Fig. 5a), with no heterogeneity (*I*^2^ = 0%).

*Coronal balance*: Four studies [16, 26, 31, 35] reported outcomes on coronal balance at final follow-up (Table 2). Patients in the PSF group achieved better coronal balance (MD = 0.68; 95%CI = 0.21–1.15; p = 0.005; Fig. 5b), but substantial heterogeneity was observed (I^2^ = 70%).

Exclusion of one study [26] focused solely on lumbar primary curves (Lenke 5–6) reduced heterogeneity (*I*^2^ = 0%) without affecting significance (MD = 0.50; 95%CI = 0.30–0.69; *p* < 0.00001; Fig. 5c).

*Thoracic kyphosis:* Five studies [28, 30, 31, 35, 36] assessed thoracic kyphosis for primary thoracic curves at the last follow-up (Table 2). Pooled analysis revealed no significant difference between groups (SMD = −0.25; 95%CI = −0.60–0.09; *p* = 0.15; Fig. 5d), with substantial heterogeneity (*I*^2^ = 58%).

Subgroup analysis of studies reporting on T5-T12 kyphosis revealed no significant difference (MD = −1.40; 95%CI = −3.21–0.42; *p* = 0.13; Fig. 5e) and reduced heterogeneity (*I*^2^ = 37%; *p* = 0.21). A separate analysis for T2-T12 kyphosis was not feasible due to the limited number of studies.

*Lumbar lordosis*: Three studies [28, 30, 36] assessed postoperative lumbar lordosis for primary thoracic curves (Table 2). Pooled analysis showed no significant difference between groups (MD = −1.89; 95%CI = −3.99–0.20; *p *= 0.08; Fig. 5f), with no heterogeneity (*I*^2^ = 0%).

Spinal height gain: The limited number of studies reporting on T1-S1 height difference from baseline precluded the possibility of conducting a meta-analysis on this outcome. The available data from Mathew et al. [33] and Mackey et al. [34] provided conflicting results (Table 3). Mathew et al. found greater spinal height gain in the VBT group (*p* = 0.028), while Mackey et al. reported a non-significant advantage favoring PSF group (*p* = 0.736).

**Table 3:** Patient reported and functional outcomes

First Author, year	SRS-22											
	Pain		Mental health		Self-image		Satisfaction		General function		Total	
	VBT	PSF	VBT	PSF	VBT	PSF	VBT	PSF	VBT	PSF	VBT	PSF
Newton et al. 2020	*4.4* ± *0.6*	*4.4* ± *0.4*	*4.3* ± *0.6*	*4.0* ± *0.7*	*4.1* ± *0.7*	*4.4* ± *0.6*	*4.3* ± *0.7*	*4.7* ± *0.5*	*4.3* ± *0.4*	*4.3* ± *0.4*	*4.2* ± *0.4*	*4.4* ± *0.4*
Newton et al. 2022	4.2 ± 0.4^e^	4 ± 0.6^e^	**3.9 ± 0.5**^**e**^	**4.0 ± 0.5**^**e**^	3.9 ± 0.6^e^	3.9 ± 0.7^e^	4.5 ± 0.4^e^	4.3 ± 0.5^e^	4.4 ± 0.3^e^	4.2 ± 0.4^e^	4.5 ± 0.4^e^	4.5 ± 0.5^e^
PSM subgroup	3.9 ± 0.5^e^	4 ± 0.6^e^	**3.9 ± 0.6**^**e**^	**4.1 ± 0.5**^**e**^	4.1 ± 0.4^e^	4.3 ± 0.4^e^	4.3 ± 0.4^e^	4.3 ± 0.6^e^	4.4 ± 0.3^e^	4.3 ± 0.4^e^	4.1 ± 0.4^e^	4.1 ± 0.5^e^
Pehlivanoglou et al. 2021	4.9 ± 0.7^a^	4.1 ± 0.7^a^	4.9 ± 0.9^a^	3.9 ± 0.9^a^	4.8 ± 1.4^a^	3.3 ± 1.4^a^	4.9 ± 1.2^a^	3.6 ± 1.2^a^	4.8 ± 0.7^a^	4.1 ± 0.7^a^	4.9 ± 1^a^	3.8 ± 1^a^
Yücekul et al. 2021	4.6 ± 0,6	4.3 ± 0,5	3.9 ± 0.8	3.4 ± 0.7	4.2 ± 0.6	4.1 ± 0.6	4.8 ± 0.4	4.3 ± 0.9	4,7 ± 0.4	4.3 ± 0.6	4.4 ± 0.5	4 ± 0.4
Maksimovic et al. 2022	NR	NR	NR	NR	NR	NR	NR	NR	NR	NR	NR	NR
Pahys et al. 2022	*4.3* ± *0.9*^*f*^	*4.3* ± *0.9*^*f*^	*4.1* ± *1.9*^*f*^	*3.9* ± *2.3*^*f*^	*4.2* ± *0.8*^*f*^	*4.4* ± *0.7*^*f*^	*4.2* ± *2.7*^*f*^	*4.6* ± *3*^*f*^	*4.6* ± *0.6*^*f*^	*4.6* ± *0.6*^*f*^	*4.3* ± *0.7*^*f*^	*4.3* ± *1.2*^*f*^

Trunk motion (Total/thoracic/thoracolumbar-lumbar)							
Flexion		Extension		Side bending		Axial rotation	
VBT	PSF	VBT	PSF	VBT	PSF	VBT	PSF
NR	NR	NR	NR	NR	NR	NR	NR
NR	NR	NR	NR	NR	NR	NR	NR
NR	NR	NR	NR	NR	NR	NR	NR
NR	NR	NR	NR	NR	NR	NR	NR
NR	NR	NR	NR	NR	NR	NR	NR
78.2 ± 18.6^a^	58.1 ± 18.6^a^	34.6 ± 14.1^a^	19.4 ± 14.1^a^	34.4 ± 14.9^a^	18.3 ± 14.9^a^	45.4 ± 19.7^a^	24.1 ± 19.7^a^
NR	NR	NR	NR	NR	NR	NR	NR
77.4 ± 21.7^§^	70.5 ± 19.2^§^	NR	NR	61.6 ± 14.3^§,g^	57.5 ± 16.3^§,g^	66.2 ± 33.1^§,g^	40.4 ± 12.3^§,g^
29.4 ± 16.1^§^	20.7 ± 9.3^§^	NR	NR	40.2 ± 11.5^§,g^	22.5 ± 10.2^§,g^	43.1 ± 26^§,g^	19.6 ± 6^§,g^
58.8 ± 8.7^§^	51.5 ± 17.6^§^	NR	NR	39.5 ± 16.7^§,g^	57.5 ± 5.6^§,g^	30.8 ± 6.1^§,g^	26.6 ± 9.9^§,g^
76.8 ± 12.3^c^	62.3 ± 16.3^c^	36 ± 13.8^c^	24.6 ± 11.9^c^	69.1 ± 15.1^c^	55.3 ± 15.7^c^	65 ± 19.1^c^	42.2 ± 18.8^c^
14.3 ± 8.7^c^	13.5 ± 7.8^c^	14 ± 8.9^c^	2 ± 5.9^c^	28.5 ± 9^c^	18.3 ± 9.4^c^	59.6 ± 18.9^c^	31.1 ±17.2^c^
62.5 ± 9.2^c^	49.5 ± 15.4^c^	22.8 ± 1^c^	22.4 ± 11.1^c^	38.7 ± 8.4^c^	36.4 ± 10.7^c^	5.2 ± 7.9^c^	9.9 ± 5.7^c^

Continuous data are displayed in Mean ± SD; *NR* Not reported; Values in bold indicate a statistically significant difference in preoperative scores between groups;  Values in italic correspond to the absence of preoperative scores between groups.

^§^Data were calculated through figures in the published study, using PlotDigitizer (https://plotdigitizer.com/)

^a^SD was calculated from the mean and p between groups using the formula from the Cochrane handbook, Sect. 7.7.3.3

^c^Combined mean and SD were calculated using the formula from the Cochrane handbook, Sect. 6.5.2.10

^e^Mean and SD were calculated from Median and range using the formula from the study by Wan, X. et al

^f^SD for each SRS-22/LIV subgroup pair was calculated from the mean and p between subgroups using the formula from the Cochrane handbook, Sect. 7.7.3.3. Next the combined mean and SD of each SRS-22 subgroup was calculated from the mean SD and n of LIV subgroups using the formula given in Cochrane handbook, chapter 6.5.2.10

^g^SD Total ROM was calculated as the sum of left and right movements. SD was derived using the variance sum law for correlated variables, assuming *r* = 0.8 due to statistical symmetry reported in motion range between right and left side (*p* > 0.05)

### Patient-reported outcomes

**Fig. 6:**
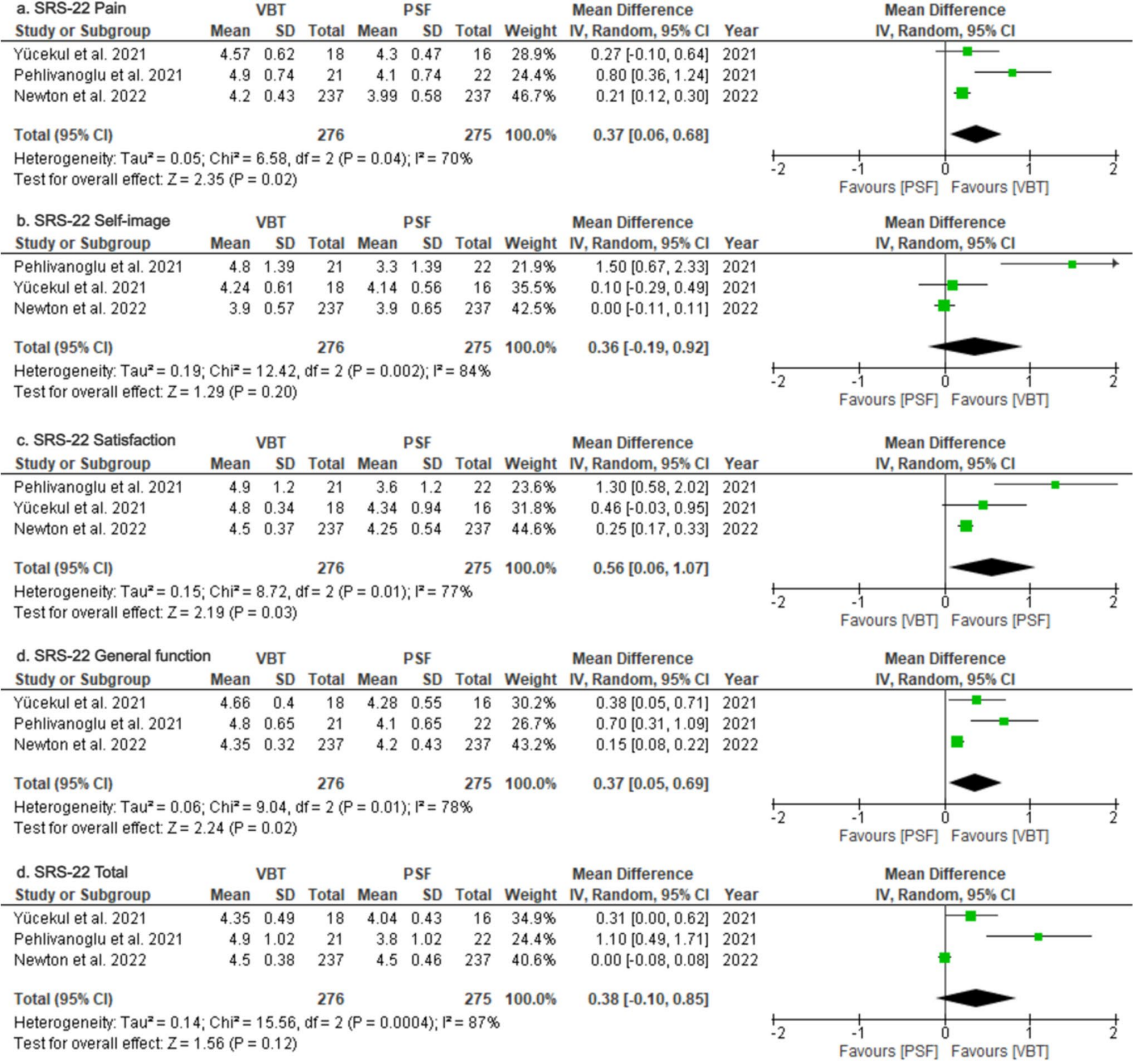
Forest Plots for SRS-22 Patient-Reported Outcomes **a** Pooled analysis comparing SRS-22 pain scores. **b** Pooled analysis comparing SRS-22 self-image scores. **c** Pooled analysis comparing SRS-22 satisfaction scores. **d** Pooled analysis comparing SRS-22 general function scores. **e** Pooled analysis comparing total SRS-22 scores

*SRS-22*: Three studies [16, 30, 36] reported outcomes for five of the six domains of the SRS-22 scale and two studies [16, 36] assessed the mental health domain. (Table 3). Thus, quantitative analysis was possible only for five of the six domains. At final follow-up the VBT group achieved better scores in pain (MD = 0.37; 95%CI = 0.06–0.68; *p* = 0.02; Fig. 6a), satisfaction (MD = 0.56; 95%CI = 0.06–1.07; *p* = 0.03; Fig. 6c) and general function (MD = 0.37; 95%CI = 0.05–0.69; *p* = 0.02; Fig. 6d), while no significant differences were found in Self-image (MD = 0.36; 95%CI = −0.19–0.92; *p* = 0.20; Fig. 6b) and total score (MD = 0.38; 95%CI = −0.10–0.85; p = 0.12; Figure e). On all outcomes, a random effects model was employed due to substantial or considerable heterogeneity (I^2^ = 70–87%). Finally, for mental health, both studies reported better outcomes for the VBT group, though Yucekul et al. [36] found the difference non-significant (*p* < 0.001 and *p* < 0.091, respectively).

### Functional outcomes – Trunk motion

**Fig. 7:**
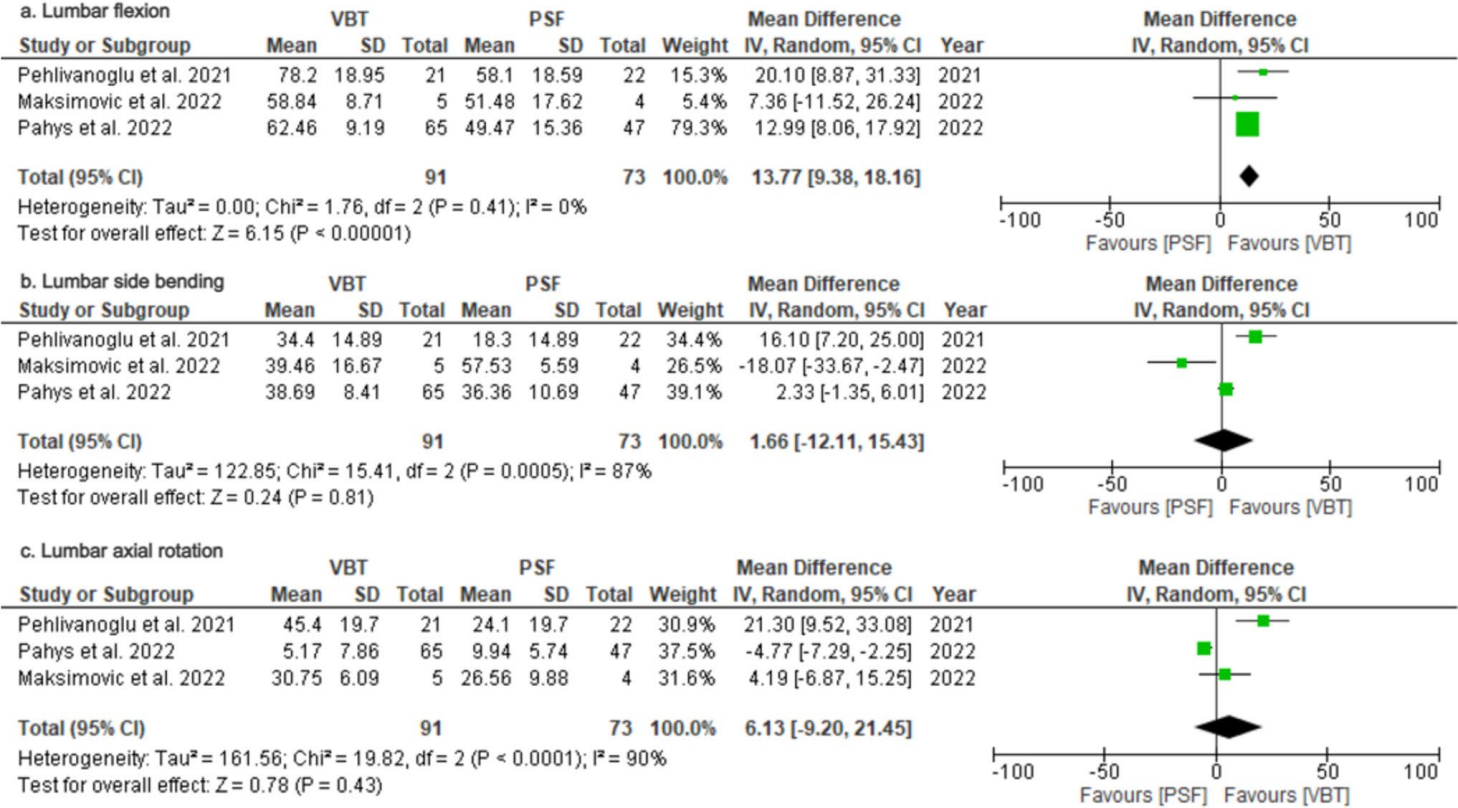
Forest Plots for Trunk Motion Outcomes. **a** Pooled analysis comparing lumbar flexion between VBT and PSF. **b** Pooled analysis comparing lumbar side bending between VBT and PSF. **c** Pooled analysis comparing lumbar axial rotation between VBT and PSF

In our analysis of trunk motion, lumbar flexion, side bending, and axial rotation were reported in three studies [14–16], permitting formal meta-analysis. Lumbar extension, thoracic and total flexion, side bending and axial rotation motions were only available in two studies each and thus summarized descriptively (Table 3). Pooled data showed greater lumbar flexion in the VBT group (MD = 13.77; 95%CI = 9.38–18.16; *p* < 0.00001; Fig. 7a), with low heterogeneity (I^2^ = 0%); whereas lumbar side bending (MD = 1.66; 95%CI = −12.11–15.43; *p* = 0.81; Fig. 7b) and axial rotation (MD = 6.13; 95%CI = −9.20–21.45; *p* = 0.43; Fig. 7c) were similar between groups, with considerable heterogeneity(*I*^2^ = 87 and *I*^2^ = 90%, respectively). For lumbar extension, one study [16] favored VBT (*p* = 0.0004), while another [15] found no difference (*p* = 0.86).

Regarding thoracic motion, no significant differences were observed in flexion between the groups in either study (Pahys: *p* = 0.63; Maksimovic: *p* = 0.31), whereas thoracic side bending was greater in the VBT group (Pahys: *p* < 0.00001; Maksimovic *p* = 0.01). Thoracic axial rotation results were inconsistent, with one study [15] favoring VBT (*p* < 0.0001) and the other [14] showing no difference (*p* = 0.05). One study [15] reported on thoracic extension and found greater motion range for VBT (*p* < 0.00001).

For total ROM, one study [15] reported greater flexion, side bending, and axial rotation in the VBT group (all *p* < 0.00001), while another [14] found no significant differences (*p* = 0.61, *p* = 0.69, *p* = 0.11). Finally, total extension in one study [15] favored the VBT group (*p* < 0.00001). These findings suggest that VBT may offer improved functional outcomes in specific motion parameters, mainly in the sagittal plane, such as lumbar flexion, though limited data for certain measures necessitate cautious interpretation.

### Perioperative outcomes

**Fig. 8:**
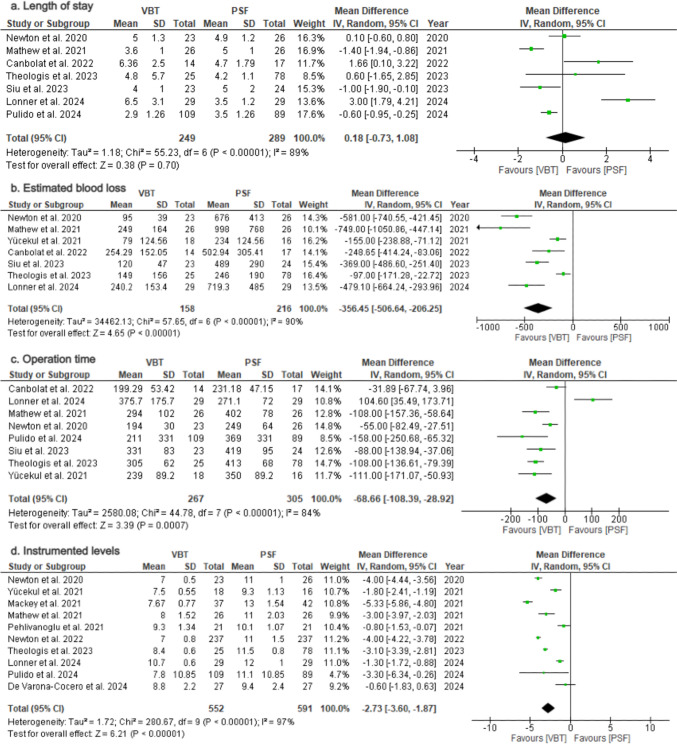
Forest Plots for Perioperative Outcomes **a** Pooled analysis comparing length of hospital stay. **b** Pooled analysis comparing estimated blood loss. **c** Pooled analysis comparing operation time. **d** Pooled analysis comparing the number of instrumented levels

*Length of stay*: Seven studies [23, 25, 28, 31–33, 35] evaluated length of hospital stay (Table 4). Pooled analysis revealed no difference between groups (MD = 0.18; 95%CI = −0.73–1.08; *p* = 0.70; Fig. 8a), with considerable heterogeneity (*I*^2^ = 89%).

**Table 4:** Perioperative outcomes and adverse events

First Author, year	LOS (days)		Blood loss (cc)		Instrumented levels		Operation time (min)		Complications		Revisions	
	VBT	PSF	VBT	PSF	VBT	PSF	VBT	PSF	VBT	PSF	VBT	PSF
Newton et al. 2020	5.0 ± 1.3	4.9 ± 1.2	95 ± 39	676 ± 413	7 ± 0.5	11 ± 1	194 ± 30	249 ± 64	10/23	0/26	7/23	0/26
Newton et al. 2022	NR	NR	NR	NR	7 ± 0.8	11 ± 1.5	NR	NR	NR	NR	38/237	3/237
Oeding et al. 2023	NR	NR	NR	NR	NR	NR	NR	NR	NR	NR	4/10	1/12
Pehlivanoglou et al. 2021	NR	NR	NR	NR	9.3 ± 1.3^b^	10.1 ± 1.1^b^	NR	NR	NR	NR	NR	NR
Pulido et al. 2024	2.9 ± 1.3^b^	3.5 ± 1.3^b^	NR	NR	7.8 ± 10.9^a^	11.1 ± 10.9^a^	211 ± 331^a^	369 ± 331^a^	NR	NR	NR	NR
Bauer et al. 2024	NR	NR	NR	NR	NR	NR	NR	NR	7/24	2/24	7/24	1/24
De Varona-Cocero et al. 2024	NR	NR	NR	NR	9.3 ± 2	9.6 ± 2.3	NR	NR	2/49	2/50	1/49	2/50
Lonner et al. 2024	6.5 ± 3.1	3.5 ± 1.2	240.2 ± 153.4	719.3 ± 485	10.7 ± 0.6	12 ± 1	375.7 ± 175.7	271.1 ± 72	4/29	0/29	2/29	0/29
Mackey et al. 2021	NR	NR	NR	NR	7.7 ± 0.8^c^	13 ± 1.5^c^	NR	NR	15/37	9/42	10/37	4/42
Yücekul et al. 2021	NR	NR	79 ± 124.6^b^	234 ± 124.6^b^	7.5 ± 0.6^b^	9.3 ± 1.1^b^	239 ± 89.2^b^	350 ± 89.2^b^	NR	NR	NR	NR
Siu et al. 2023	4 ± 1	5 ± 2	120 ± 47	498 ± 290	NR	NR	331 ± 83	419 ± 95	NR	NR	9/23	4/24
Canbolat et al. 2022	6.36 ± 2.5	4.7 ± 1.8	254.3 ± 152.1	502.9 ± 305.4	NR	NR	199.3 ± 53.4	231.2 ± 47.2	4/14	5/17	NR	NR
Theologis et al. 2023	4.8 ± 5.7	4.2 ± 1.1	149 ± 156	246 ± 190	8.4 ± 0.6	11.5 ± 0.8	305 ± 62	413 ± 68	5/25	9/78	5/25	6/78
Mathew et al. 2021	3.6 ± 1	5 ± 1	249 ± 164	998 ± 768	8 ± 1.5	11 ± 2	294 ± 102	402 ± 78	3/26	3/26	2/26	2/26

Continuous data are displayed in Mean ± SD; *NR* Not reported

^a^SD was calculated from the mean and p between groups using the formula from the cochrane handbook, Sect. 7.7.3.3

^d^SD was calculated from range and N using the formula from the study by Walter SD, Yao X

^b^Mean and SD were calculated from Median and IQR using the formula from the study by Wan, X. et al

*Estimated blood loss*: Seven studies [23, 28, 31–33, 35, 36] assessed intraoperative blood loss (Table 4). Pooled results indicated significantly lower blood loss in the VBT group (MD = −356.45; 95%CI = −506.64–−206.25; *p* < 0000.1; Fig. 8b), with considerable heterogeneity (*I*^2^ = 90%).

*Operation time*: Eight studies [23, 25, 28, 31–33, 35, 36] examined operation time (Table 4). Significantly lower operation time was reported in the VBT group (MD = −68.66; 95%CI = −108.39–−28.90; p = 0,0007; Fig. 8c), with considerable heterogeneity (*I*^2^ = 84%).

*Instrumented levels*: Nine studies [16, 24, 25, 28, 30, 32–34, 36] reported outcomes in the instrumented levels (Table 4). VBT group required shorter instrumentations compared to PSF group (MD = −2.73; 95%CI = −3.60–−1.87; *p* < 0000.1; Fig. 8d), with considerable heterogeneity (*I*^2^ = 97%).

### Adverse events

**Fig. 9:**
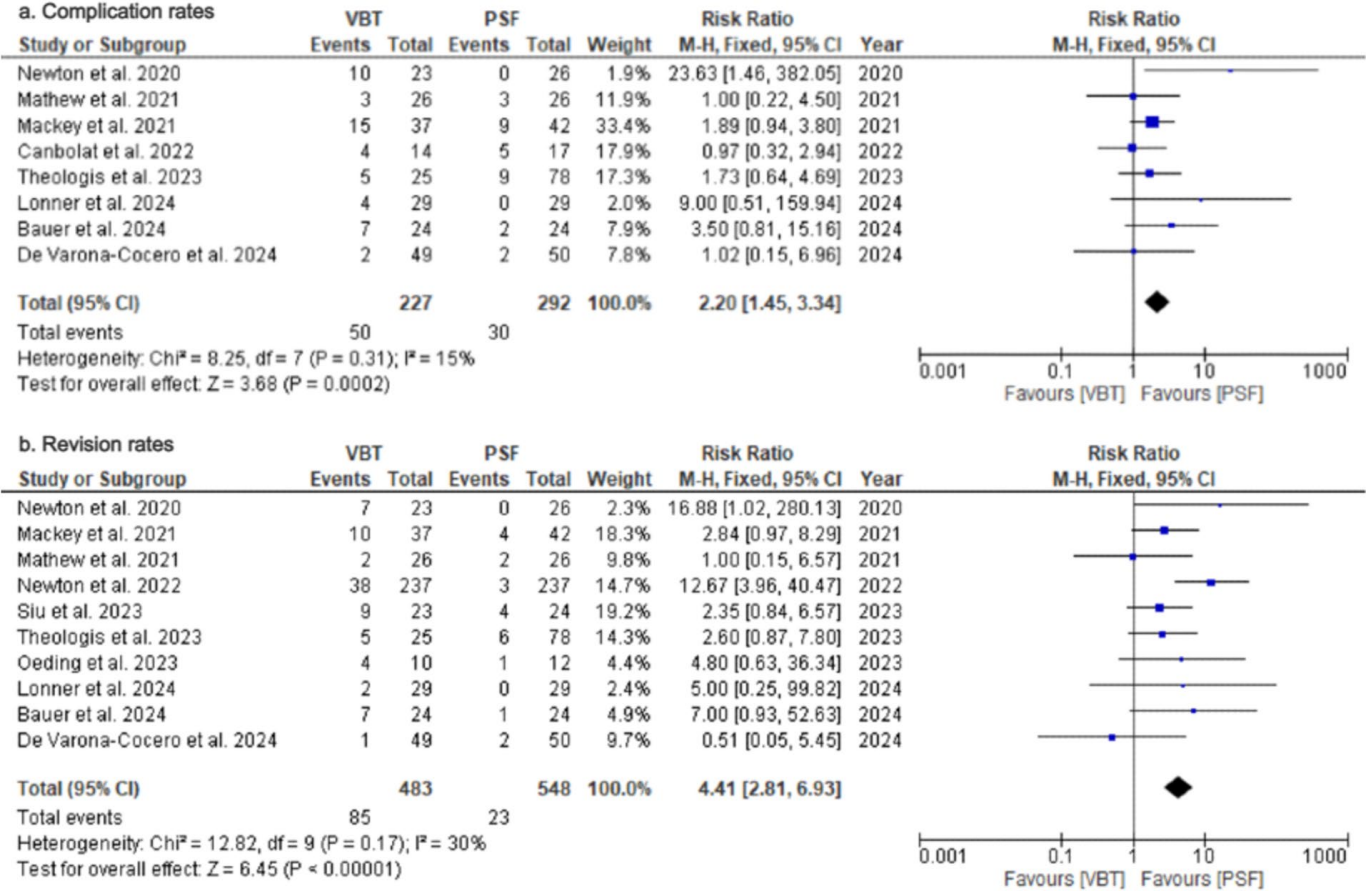
Forest Plots for Complication and Revision Rates. **a** Pooled analysis comparing complication rates between VBT and PSF. **b** Pooled analysis comparing revision rates between VBT and PSF

*Complication rates*: Eight studies [23, 24, 26, 28, 32–35] assessed overall complication rates at final follow-up (Table 4). The VBT group had significantly higher complication rate compared to PSF group (RR = 2.20; 95%CI = 1.45–3.34; *p* = 0.0002; Fig. 9a), with low heterogeneity (*I*^2^ = 15%).

*Revision rate*: Ten studies [24, 26, 28–35] reported outcomes on revision rates at final follow-up (Table 4). The VBT group had a significantly higher reoperation risk than the PSF group (RR = 4.41; 95%CI = 2.81–6.93; *p* < 0000.1; Fig. 9b), with low heterogeneity (I2 = 30%; *p* = 0.17).

### Sensitivity analysis

A RoB sensitivity analysis was performed to evaluate the robustness of the results. This involved the exclusion of studies with a high RoB to assess whether their occlusion had a disproportionate impact on the overall results. The analysis confirmed that the exclusion of these studies did not alter the statistical significance of the primary and secondary outcomes.

### Publication bias

**Fig. 10:**
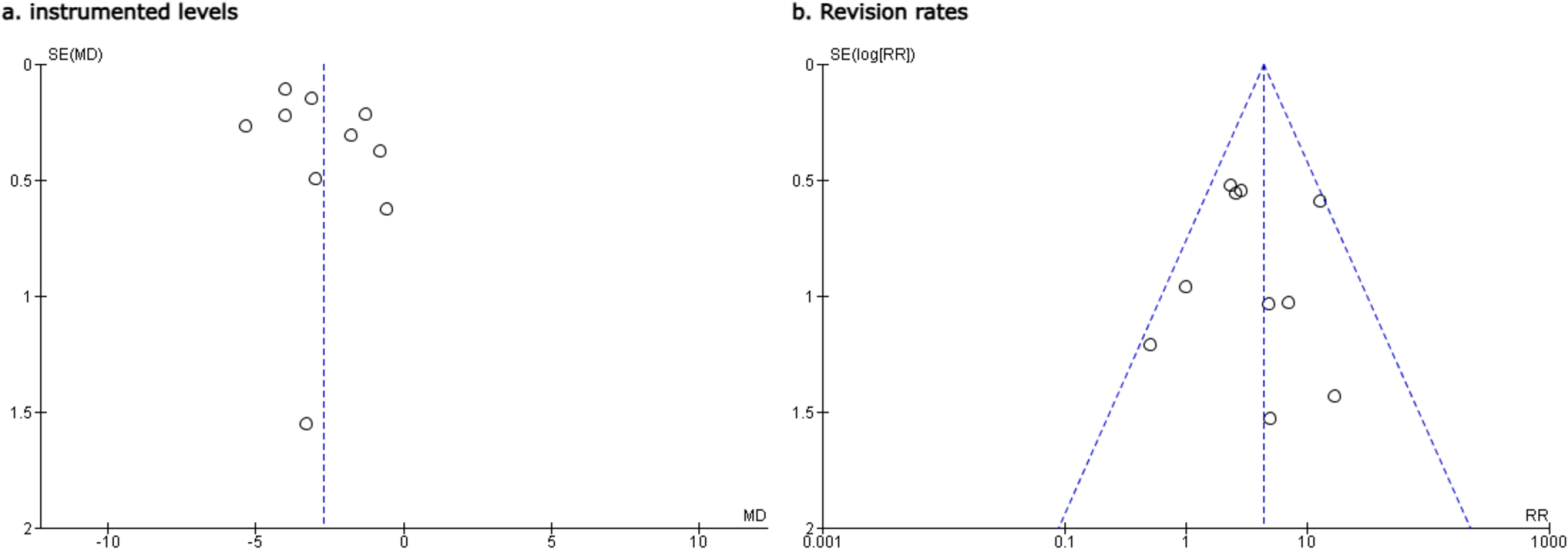
Funnel plots assessing potential publication bias in the analysis of **a** instrumented levels and **b** revision rates. Each point represents a study included in the meta-analysis. The symmetry of the plot suggests a low likelihood of publication bias for both outcomes

The funnel plots for the outcomes instrumented levels and revision rates displayed a symmetrical distribution of effect sizes around the mean, suggesting a low likelihood of publication bias (Fig. 10).

## Discussion

While both techniques achieved acceptable correction [38], PSF group had significantly lower primary and secondary curve angles, with some studies reporting a twofold correction over VBT. This aligns with the biomechanical principles of spinal fusion, which provides rigid stabilization and greater corrective force. In contrast, VBT offers dynamic correction over time via growth modulation [39] in addition to the initial intraoperative correction, potentially diminishing these differences in longer follow-ups [40].

Shoulder balance is a key determinant of patient satisfaction. Terheyden et al. identified several parameters that can influence shoulder balance, including significant Cobb angle correction of the distal thoracic curve [41]. In our analysis, VBT patients achieved a lower postoperative shoulder height difference. This could be attributed to PSF achieving greater intraoperative curve correction, while VBT provides a more gradual, controlled correction through its dynamic, growth-modulating mechanism, allowing for continuous spinal adjustments over time. This process promotes more natural shoulder symmetry, reducing the likelihood of postoperative shoulder imbalance.

Coronal balance is essential for spinal harmony, as it directly impacts spinal alignment, posture and overall patient satisfaction [42]. Failure to correct coronal balance after surgery can result in increased stress on spinal structures and accelerate degenerative changes [43]. Additionally, coronal imbalance can manifest as waist asymmetry, affecting patient’s self-image [26, 38]. Our analysis of five studies showed that, while both techniques achieve acceptable coronal balance, PSF provides better coronal alignment at two-year follow-up.

Idiopathic scoliosis is commonly associated with alterations in the sagittal profile, particularly hypokyphosis in primary thoracic curves, which is a structural characteristic of the deformity and independent of pelvic parameters [44]. Baroncini et al. previously reported favorable outcomes on VBT’s potential to improve thoracic kyphosis [45]. However, our analysis found no statistically statistical difference in postoperative thoracic kyphosis between groups for primary thoracic curves, while some studies noted marginal hypokyphosis in the VBT group [30, 35]. Similarly, no difference was observed in lumbar lordosis, except for one study reporting marginal hyperlordosis in the PSF group [28].

The two-year follow-up analysis of SRS-22 domains showed that VBT led to superior outcomes in pain reduction, satisfaction and general function compared to PSF, indicating notable early postoperative quality-of-life benefits. However, no significant differences were observed in self-image, or total scores. The improved general function in VBT may be attributed to the motion-preserving nature of this technique. Similarly, pain scores could be attributed to the reduced mechanical stress on adjacent segments and the prevention of stiffness-related discomfort due to preserved spinal mobility [46]. This aligns with previous studies reporting lower incidence of persistent pain after VBT [46, 47]. Finally, higher satisfaction in the VBT group may be linked to factors such as pain relief, improved appearance, enhanced physical function and the less invasive nature of the procedure [48].

One of the most significant advantages of VBT over PSF is its ability to preserve spinal motion [1]. Our analysis demonstrated that VBT patients had significantly greater lumbar flexion; however, no significant differences were observed between groups in lumbar side bending or axial rotation. This aligns with previous reports on spinal motion in patients treated with VBT [7]. The lack of difference in side bending may be attributed to several factors. Lateral flexion relies heavily on the flexibility of intervertebral discs and facet joints, which may still be affected by tether tension and postoperative soft tissue adaptations in VBT patients. Nicolini et al. [49] found in a finite element analysis study that tether tension directly affects the residual ROM, primarily lateral bending. Additionally, PSF patients may develop compensatory mechanisms at adjacent, non-fused segments, preserving a degree of lateral bending that reduces the observable difference between groups.

Pooled results from this meta-analysis indicate that VBT is associated with shorter operative times and significantly less intraoperative blood loss. This is likely due to the less invasive nature of this procedure and the need for shorter instrumentations compared to PSF. However, hospitalization time was similar between groups. These perioperative benefits may translate into a faster initial recovery, though this does not  seem to significantly impact the length of hospital stay.

Regarding spinal height gain from baseline, the two studies reported conflicting results. The favorable outcome for PSF in Mackey’s [34] study may be attributed to notable differences in study populations between groups, particularly the higher proportion of early-onset scoliosis patients in the PSF group with greater growth potential. These differences in patient characteristics could explain the trend favoring PSF in Mackey’s study [34].

Finally, VBT was associated with significantly higher complication and revision rates. The high reoperation risk, often due to tether breakage or overcorrection, represents a major limitation of VBT. In contrast, PSF displayed greater long-term stability, with lower complication and revision rates. This trade-off between motion preservation and durability should be carefully weighted when selecting the optimal treatment for each patient.

This meta-analysis offers several strengths, including adherence to PRISMA guidelines, a comprehensive search strategy across multiple databases, strict inclusion criteria, rigorous data extraction and RoB assessment performed by independent reviewers. The inclusion of both English and non-English studies minimizes the risk of language bias. Additionally, the use of sensitivity and subgroup analyses helps to address heterogeneity and improve the reliability of the results.

However, we should also acknowledge several limitations. The majority of the included studies were retrospective, which introduces potential selection and reporting biases. The follow-up duration for most studies was limited to two years, which may not fully capture long-term outcomes, particularly for VBT, which relies on growth modulation over time. Most studies focus on idiopathic scoliosis, while early-onset scoliosis patients, with greater growth potential, may achieve better results with VBT; data on this subgroup remain limited. Additionally, some outcomes had a limited number of studies, reducing the strength of the conclusions that can be drawn. In some cases, imputation of missing data was required to enable pooled analyses. Although we applied validated methods, imputation inherently introduces the risk of measurement bias. Finally, the heterogeneity among included studies in terms of surgical techniques, follow-up durations, and outcome measures may also affect the comparability of the results.

Although these factors may reduce the robustness of our results and should be taken into account when interpreting them, our meta-analysis synthesizes all the available evidence and also highlights gaps to guide clinical decisions and future research.

## Conclusion

This meta-analysis provides a comprehensive comparison of VBT and PSF for the treatment of idiopathic scoliosis. While PSF demonstrates superior curve correction, coronal alignment and lower complication and revision rates, VBT better preserves spinal motion, promotes better shoulder symmetry, reduces postoperative pain, and improves early postoperative function and quality of life. Additionally, VBT requires shorter instrumentations. Both techniques have distinct advantages and limitations, and the selection of surgical technique should be individualized based on patient-specific factors, including skeletal maturity, curve characteristics, and surgeon's clinical judgment and preference. Long-term studies are necessary to further elucidate the durability of VBT outcomes and to optimize surgical decision-making in managing idiopathic scoliosis.

## Supplementary Information

Below is the link to the electronic supplementary material.

Supplementary file1 (DOCX 35 KB)

Supplementary file2 (DOCX 16 KB)

## Data Availability

The data supporting the findings of this study are available from the corresponding author upon reasonable request
