# Supplementary material for: Comparative meta-analysis of vertebral body tethering and posterior spinal fusion in patients with idiopathic scoliosis. Evaluation of radiographic, perioperative, clinical, patient-reported outcomes, and complication rates
Source: Spine Deform. 2025 Jun 8;13(5):1399–420. doi: 10.1007/s43390-025-01113-z (PMC12402045; doi:10.1007/s43390-025-01113-z)
Supplement: Supplementary file 2 — Supplementary file2 (DOCX 16 KB) [file 43390_2025_1113_MOESM2_ESM.docx]

**Article title:** Comparative Meta-Analysis of Vertebral Body Tethering and Posterior Spinal Fusion in patients with Idiopathic Scoliosis: Evaluation of radiographic, perioperative, clinical, patient-Reported Outcomes, and complication Rates

**Journal name:** Spine Deformity

**Author names:** Stavros Stamiris, Cornelius Sofos, Athanasios Sarridimitriou, Panagiotis Kakoulidis, Panagiotis Christidis, Dimitrios Stamiris, Elissavet Anestiadou, Angeliki Cheva, Xristiana Xatzianestiadou, Pavlos Christodoulou, Christos Karampalis

**Corresponding author**

**Name:** Stamiris Stavros

**Affiliation:** Department of Orthopaedics, 424 General Military Hospital, Thessaloniki, Greece, 3^rd^ Academic Department of Orthopedics, Faculty of Medicine, Aristotle University of Thessaloniki

**E-mail Address:** [st.stamiris@hotmail.com](mailto:st.stamiris@hotmail.com)

| **Supplementary Table 2. Search string used in each database** | |
| --- | --- |
| **Database** | **Search string** |
| **Pubmed** | ("Idiopathic scoliosis" OR "Adolescent idiopathic scoliosis" OR "AIS" OR "Early onset scoliosis" OR "EOS" OR "Juvenile idiopathic scoliosis" OR "JIS" OR "infantile idiopathic scoliosis" OR "IIS") AND ("Posterior spinal fusion" OR "PSF" ΟR "posterior spinal instrumentation and fusion" OR "PSIF" OR "PI" OR "posterior instrumentation" OR "spinal fusion") AND ("Vertebral body tethering" OR "AVBT" OR "VBT" OR "AST" OR "Anterior Spinal tethering" OR "posterior lumbar spine tethering" OR "PLST") AND ("Major cobb" OR "Main curve" OR "Major curve" OR "major curve magnitude" OR "Scoliosis angle" OR "Cobb angle" OR "Primary curve" OR "Curve magnitude" OR "Minor cobb" OR "Secondary curve" OR "Minor curve" OR "Coronal balance" OR "Coronal imbalance" OR "C7-central Sacral vertical line" OR "C7-CSVL" OR "Trunk shift" OR "T1-S1 height" OR “Spinal height” OR "Shoulder height difference" OR "Shoulder height" OR "sagittal kyphosis" OR "T5–T12 kyphosis" OR "T2–T12 kyphosis" OR "Thoracic Kyphosis" OR "Kyphosis" OR "Kyphosis angle" OR "Lumbar lordosis" OR "Fused levels" OR "trunk motion" OR "ROM" OR "Range of motion" OR "Sagittal motion" OR "coronal motion" OR "flexion" OR "extension" OR "lateral bending" OR "side-bending" OR "rotation" OR "axial twisting" OR "SRS-22" OR "Scoliosis Research Society-22" OR "LOS" OR "Length of stay" OR "Hospital stay" OR "Blood loss" OR "Amount of Bleeding" OR "Bleeding" OR "EBL" OR "Operation time" OR "Surgery Duration" OR "Case length" OR "Operation duration" OR "OR time" OR "Operative time" OR "Revision rate" OR "Revision surger*" OR "complications" OR "Revision") |
| **Scopus** | TITLE-ABS-KEY(("Idiopathic scoliosis" OR "Adolescent idiopathic scoliosis" OR "AIS" OR "Early onset scoliosis" OR "EOS" OR "Juvenile idiopathic scoliosis" OR "JIS" OR "Infantile idiopathic scoliosis" OR "IIS")  AND ("Posterior spinal fusion" OR "PSF" OR "Posterior spinal instrumentation and fusion" OR "PSIF" OR "PI" OR "Posterior instrumentation" OR "Spinal fusion")  AND ("Vertebral body tethering" OR "AVBT" OR "VBT" OR "AST" OR "Anterior spinal tethering" OR "Posterior lumbar spine tethering" OR "PLST") AND ("Major Cobb" OR "Main curve" OR "Major curve" OR "Major curve magnitude" OR "Scoliosis angle" OR "Cobb angle" OR "Primary curve" OR "Curve magnitude" OR "Minor Cobb" OR "Secondary curve" OR "Minor curve" OR "Coronal balance" OR "Coronal imbalance" OR "C7-central sacral vertical line" OR "C7-CSVL" OR "Trunk shift" OR "T1-S1 height" OR "Spinal height" OR "Shoulder height difference" OR "Shoulder height" OR "Sagittal kyphosis" OR "T5–T12 kyphosis" OR "T2–T12 kyphosis" OR "Thoracic kyphosis" OR "Kyphosis" OR "Kyphosis angle" OR "Lumbar lordosis" OR "Fused levels" OR "Trunk motion" OR "ROM" OR "Range of motion" OR "Sagittal motion" OR "Coronal motion" OR "Flexion" OR "Extension" OR "Lateral bending" OR "Side-bending" OR "Rotation" OR "Axial twisting" OR "SRS-22" OR "Scoliosis Research Society-22" OR "LOS" OR "Length of stay" OR "Hospital stay" OR "Blood loss" OR "Amount of bleeding" OR "Bleeding" OR "EBL" OR "Operation time" OR "Surgery duration" OR "Case length" OR "Operation duration" OR "OR time" OR "Operative time" OR "Revision rate" OR "Revision surger*" OR "Complications" OR "Revision")) |
| **Web of Science** | TS=("Idiopathic scoliosis" OR "Adolescent idiopathic scoliosis" OR "AIS" OR "Early onset scoliosis" OR "EOS" OR "Juvenile idiopathic scoliosis" OR "JIS" OR "Infantile idiopathic scoliosis" OR "IIS")  AND TS=("Posterior spinal fusion" OR "PSF" OR "Posterior spinal instrumentation and fusion" OR "PSIF" OR "PI" OR "Posterior instrumentation" OR "Spinal fusion") AND TS=("Vertebral body tethering" OR "AVBT" OR "VBT" OR "AST" OR "Anterior spinal tethering" OR "Posterior lumbar spine tethering" OR "PLST") AND TS=("Major Cobb" OR "Main curve" OR "Major curve" OR "Major curve magnitude" OR "Scoliosis angle" OR "Cobb angle" OR "Primary curve" OR "Curve magnitude" OR "Minor Cobb" OR "Secondary curve" OR "Minor curve" OR "Coronal balance" OR "Coronal imbalance" OR "C7-central sacral vertical line" OR "C7-CSVL" OR "Trunk shift" OR "T1-S1 height" OR "Spinal height" OR "Shoulder height difference" OR "Shoulder height" OR "Sagittal kyphosis" OR "T5–T12 kyphosis" OR "T2–T12 kyphosis" OR "Thoracic kyphosis" OR "Kyphosis" OR "Kyphosis angle" OR "Lumbar lordosis" OR "Fused levels" OR "Trunk motion" OR "ROM" OR "Range of motion" OR "Sagittal motion" OR "Coronal motion" OR "Flexion" OR "Extension" OR "Lateral bending" OR "Side-bending" OR "Rotation" OR "Axial twisting" OR "SRS-22" OR "Scoliosis Research Society-22" OR "LOS" OR "Length of stay" OR "Hospital stay" OR "Blood loss" OR "Amount of bleeding" OR "Bleeding" OR "EBL" OR "Operation time" OR "Surgery duration" OR "Case length" OR "Operation duration" OR "OR time" OR "Operative time" OR "Revision rate" OR "Revision surger*" OR "Complications" OR "Revision") |
| **Cochrane** | ("Idiopathic scoliosis" OR "Adolescent idiopathic scoliosis" OR "AIS" OR "Early onset scoliosis" OR "EOS" OR "Juvenile idiopathic scoliosis" OR "JIS" OR "Infantile idiopathic scoliosis" OR "IIS") AND ("Posterior spinal fusion" OR "PSF" OR "Posterior spinal instrumentation and fusion" OR "PSIF" OR "PI" OR "Posterior instrumentation" OR "Spinal fusion") AND ("Vertebral body tethering" OR "AVBT" OR "VBT" OR "AST" OR "Anterior spinal tethering" OR "Posterior lumbar spine tethering" OR "PLST") AND ("Major Cobb" OR "Main curve" OR "Major curve" OR "Major curve magnitude" OR "Scoliosis angle" OR "Cobb angle" OR "Primary curve" OR "Curve magnitude" OR "Minor Cobb" OR "Secondary curve" OR "Minor curve" OR "Coronal balance" OR "Coronal imbalance" OR "C7-central sacral vertical line" OR "C7-CSVL" OR "Trunk shift" OR "T1-S1 height" OR "Spinal height" OR "Shoulder height difference" OR "Shoulder height" OR "Sagittal kyphosis" OR "T5–T12 kyphosis" OR "T2–T12 kyphosis" OR "Thoracic kyphosis" OR "Kyphosis" OR "Kyphosis angle" OR "Lumbar lordosis" OR "Fused levels" OR "Trunk motion" OR "ROM" OR "Range of motion" OR "Sagittal motion" OR "Coronal motion" OR "Flexion" OR "Extension" OR "Lateral bending" OR "Side-bending" OR "Rotation" OR "Axial twisting" OR "SRS-22" OR "Scoliosis Research Society-22" OR "LOS" OR "Length of stay" OR "Hospital stay" OR "Blood loss" OR "Amount of bleeding" OR "Bleeding" OR "EBL" OR "Operation time" OR "Surgery duration" OR "Case length" OR "Operation duration" OR "OR time" OR "Operative time" OR "Revision rate" OR "Revision surger*" OR "Complications" OR "Revision") |
